# Could B‐scan technique compromise a promising clinical study?

**DOI:** 10.1002/cam4.6230

**Published:** 2023-06-23

**Authors:** Danilo Biondino, Isabella Fioretto, Mario Graziano

**Affiliations:** ^1^ Department of Medicine Surgery and Dentistry University of Salerno Salerno Italy

To the Editor

We read with great interest the article by Jiang et al. concerning the use of bedside ocular ultrasonography (US) for diagnosing increased intracranial pressure in patients with leptomeningeal metastases from non‐small‐cell lung cancer.^1^

Our congratulations go to the authors for their intriguing paper. However, we would also like to provide some feedback through our comments.

In this study the authors used US B‐scan with a 5–12 MHz linear array ultrasonic probe to measure optic nerve sheath diameter (ONSD) and eyeball vertical diameter (EVD).

While it is a commonly practiced non‐invasive method to detect intracranial hypertension, the accuracy of ONSD measurements through US B‐scan is often compromised by various artifacts,^2^, ^3^, ^4^ as supported by literature. It is noteworthy that B‐scan may have a high sensitivity in detecting small optic nerve (ON) calcifications such as ON drusen, but it is not entirely reliable for measurements.^5^

However one of the major pitfalls of this paper is that the scans were performed with closed eyelids. In this modality the image quality is decreased by sound attenuation due to the lid tissue, making the result even more untrustworthy.

Moreover with closed eyelids to assess if the eye is in primary position is very difficult. To examine the eye in primary position is very important for measuring both the ONSD both the anteroposterior diameter. In case of the ONSD, changes in the eye position could lead to a variation of the amount of the cerebrospinal fluid that surrounds the ON, resulting in a different diameter.

The same problem is present in case of the measurement of the anteroposterior diameter that is generally known as axial length (AL) of the eye, and that the authors call EVD.

First of all, from the anatomic point of view EVD refers to the maximal measurement of the distance between the superior and inferior poles of the eye. Instead the authors measured the distance between the anterior and posterior poles, where the anterior one is the corneal apex, which is the most prominent point on the front surface of the cornea, and the posterior shows the ON insertion, and it should be correctly named as AL.

The incorrect AL measurement due to closed eye lids is clearly shown in Figure 1 where not only the crystalline lens is not displayed, but the eyeball appears oval instead of roundish, that is an artifact due to a chord of the eye measurement and not the real diameter.

**FIGURE 1:**
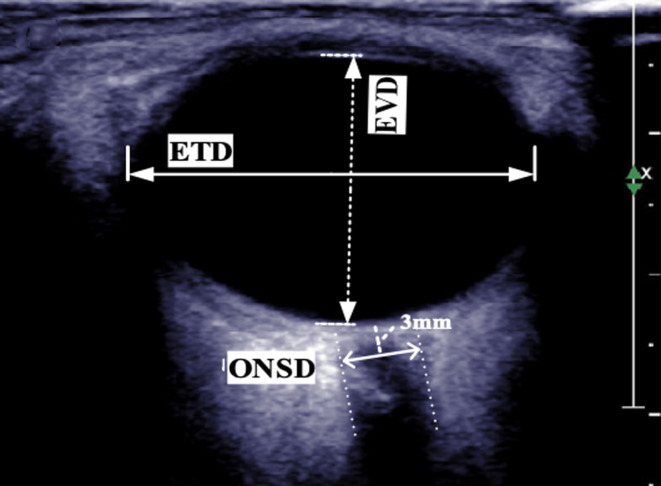
Parameters on eyeball recorded by authors in B scan echography. ETD, eyeball transverse diameter; EVD, eyeball vertical diameter; ONSD, optic nerve sheath diameter.

However, even in case of correct positioning of the probe, B‐scan is not reliable in the AL measurement because along the axis there are structures that have a different sound speed, which play an important role in determining the AL of the eyeball. In fact, only by knowing the time it takes for these ultrasound waves to pass through the ocular structures, it is possible to convert time into distance.

From this, it can be deduced how important it is, to obtain an accurate measurement, to know the speed of ultrasound in ocular structures. Most commercially available biometers use different speeds depending on the media through which they pass, which are 1532 m/s in the aqueous and vitreous humor, 1640 in the cornea, and 1661 in the lens.

Unfortunately, this does not happen with B‐scan, in fact most of use an average value of 1550 m/s for all structures passed through. These devices should no longer be used for eyeball measurements because they can cause serious errors in the anteroposterior measurement of axes, especially in patients with particularly long or short eyes.

For all these reasons we question if the use of B‐scan technique could compromise the results of a so interesting clinical study. To perform a more reliable comparison, the authors could have utilized the so‐called standardized A scanning technique which allows more objective measurements.^6^

As take‐home messages, we would like to suggest performing all the examinations with open eyelids and with the eye in the primary position after the use of anesthetic eye drops, utilizing the standardized A‐scan technique.

## AUTHOR CONTRIBUTIONS

**Danilo Biondino:** Writing – review and editing (equal). **Isabella Fioretto:** Writing – review and editing (equal). **Mario Graziano:** Writing – review and editing (equal).

## CONFLICT OF INTEREST STATEMENT

All the authors declare that they have no conflicts of interest.

## ETHICS STATEMENT

Confirm adherence to ethical guidelines and indicate ethical approvals (IRB) and use of informed consent, as appropriate.
